# Effects of Insomnia on Peptic Ulcer Disease Using Mendelian Randomization

**DOI:** 10.1155/2021/2216314

**Published:** 2021-09-25

**Authors:** Ling-Feng Zha, Jiang-Tao Dong, Jing-Lin Wang, Qian-Wen Chen, Jian-Fei Wu, Ying-Chao Zhou, Shao-Fang Nie, Xin Tu

**Affiliations:** ^1^Department of Cardiology, Union Hospital, Tongji Medical College, Huazhong University of Science and Technology, Wuhan 430022, China; ^2^Department of Cardiovascular Surgery, Union Hospital, Tongji Medical College, Huazhong University of Science and Technology, Wuhan 430022, China; ^3^Hubei Maternal and Child Health Hospital, Wuhan 430070, China; ^4^College of Life Science and Technology, Center for Human Genome Research, Cardio-X Institute, Huazhong University of Science and Technology, Wuhan 430074, China; ^5^Heart Center, Qingdao Women and Children's Hospital, Qingdao University, Qingdao 266000, China

## Abstract

**Objectives:**

Observational studies indicate that insomnia may increase risk of peptic ulcer disease (PUD). Our purpose is to clarify the possible causal relationship between insomnia and PUD by Mendelian randomization analyses.

**Methods:**

We carried out analyses using summary statistics data for genetic variants reported from a GWAS of insomnia (*N* = up to 1,331,010 individuals) and from a GWAS of PUD (*N* = up to 456,327 individuals). Three Mendelian randomization approaches were used to explore whether insomnia might play a causal role in PUD, and pathway and functional enrichment analyses were conducted to anticipate the underlying mechanisms.

**Results:**

Conventional Mendelian randomization analysis showed clear causality between insomnia and PUD; 1 SD increased insomnia incident was related to a 19% higher risk of PUD (*P* = 6.69 × 10^−16^; OR, 1.19 (95% CI, 1.14-1.24)). The associations between insomnia and PUD were consistent in the other two analyses performed using the weighted median method (*P* = 7.75 × 10^−7^; OR, 1.16 (95% CI, 1.09-1.23)) and MR-Egger regression (*P* = 5.00 × 10^−3^; OR, 1.27 (95% CI, 1.07-1.50)). Moreover, no evidence indicated a reverse causality between PUD events and insomnia symptoms. Pathway and functional enrichment analyses indicated that the mechanisms of insomnia effect on PUD may be through various ways, such as the immune system and oxidative stress.

**Conclusions:**

This Mendelian randomization study suggests insomnia as a causal risk factor for PUD. The potential mechanisms included may be immune and oxidative stress. These findings indicate that improving sleep quality could have substantial health benefits.

## 1. Introduction

Insomnia is a common mental disorder; it is by far the most common sleep disorder in medical practice. About 30% of the common population report symptoms of insomnia [1]. Insomnia is considered as a key factor referring to the occurrence and progress of chronic inflammatory diseases; it is closely associated with gastrointestinal symptoms, high blood pressure, asthma, systemic lupus erythematosus, autonomic nervous system dysfunction, impaired blood sugar control, and increased inflammation [2].

Peptic ulcer disease (PUD) is a common gastrointestinal disease involving rupture (ulceration) of the alimentary canal mucosa, mainly occurring in the gastric area and duodenum. Epidemiology shows that the annual incidence of PUD is 0.1-0.3%, and the lifetime prevalence in the general population is about 5-10% [3]. More than 4 million people suffer from the disease each year, resulting in high medical costs [4]. People with PUD have a lower quality of life. The cardinal symptoms of PUD are upper abdominal pain but also can have increasing saliva secretion, heartburn, belching, vomiting, and other gastrointestinal symptoms [5]. It is called peptic ulcer because it was thought that gastric and duodenal are included caused by the digestion of the mucous membrane itself by gastric acid and pepsin. Although the etiology of PUD is not yet clear, H. pylori infection, gastric acid overproduction, and weakened gastric mucosal protection mechanism are recognized as the main pathogenesis in recent years [3].

It is well known that sleep quality is closely related to gastrointestinal health. It is reported that the sleep quality of patients with peptic ulcer is worse than that of normal people. Evidence suggests that in older populations, PUD patients have poorer sleep quality compared to the healthy [6]. Recently, a study found that among women, those who slept less than seven hours a night had twice the risk of developing PUD compared to those who slept more than nine hours a night [7]. Another study investigated whether poor sleep quality predicted recurrence of PUD in 1,538 elderly patients who had been inoculated with H. pylori; the result shows that the recurrence rate of PUD was higher in poor sleepers than in good sleepers [8]. With the deepening of the research on peptic ulcer, scholars believe that there is a certain correlation between sleep quality and peptic ulcer, which is both the cause of the disease and the rehabilitation disorder. Studies have shown that sleep disorders (especially insomnia) can increase the incidence of PUD. Peptic ulcer patients will be due to upper abdominal pain or discomfort from sleep to wake up, occurring a variety of forms of sleep disorders, such as difficulty in falling asleep, increased wakefulness, and prolonged wakefulness. Although a growing number of clinical studies suggest a close relationship between insomnia and PUD, the causal relationship between them remains uncertain. Clarifying the causal relationship between insomnia and PUD is of great significance for understanding the pathogenesis of PUD, as well as for providing potentially new prevention strategies.

Mendelian randomization is a flourishing branch of genetic epidemiological methodology. Its core is to use genetic data as a bridge to clarify thoroughly the causality between the exposure and the outcome. MR is based on the most basic Mendelian law of heredity that parental alleles are randomly assigned to offspring and that genotype determines phenotype [9]. Since genotype is innate, it must be ahead of the time of outcome, and it is not disturbed by other confounding factors such as acquired environment, so it can be used as a powerful tool to study the causality between exposure and outcome. Compared with traditional epidemiological methods, this approach can indicate the direction of exposure and outcome, and therefore, the causal relationship between the two, not just the association [10]. The design strategy of two-sample MR is to establish two independent samples from the same population that are associated with the study population. MR of the two samples is based on long-term data from genome-wide association studies (GWASs). Given its enlarged sample size, the statistical ability is greatly improved [11]. Currently, the two-sample MR is widely used due to the large amount of public data from GWAS cooperative groups around the world.

In this study, we explored a possible causal relationship between insomnia and PUD by bidirectional Mendelian randomization analyses, using available public data from two recent large-scale GWAS for insomnia [12] and PUD [13].

## 2. Methods

### 2.1. Genetic Variants Associated with Insomnia

In the first phase analysis, 12 independent single nucleotide polymorphisms (SNPs) (*P* < 5 × 10^−8^ for GWAS significant level; *r*^2^ < 0.1 for linkage disequilibrium) that were susceptible to insomnia in the dataset (SSGAC) (113,006 participants) were included in the analysis [14]. Eight of 12 SNPs associated with insomnia were included in the PUD GWAS dataset and were selected for our instrumental variable analyses (Table 1). For our secondary phase analysis, in order to make our sensitivity analysis to achieve sufficient statistical power, another larger set of SNPs was used in our main analysis, which was retrieved from a recent GWAS for insomnia involving 1,331,010 European people [12]. In this analysis, 248 independent SNPs were significantly associated with insomnia (*P* < 5 × 10^−8^; *r*^2^ < 0.1). 242 of them were included in the PUD GWAS dataset and were chosen for the MR analysis (see supplementary Table 1). This is enough to produce a powerful genetic tool that can be used to obtain an unbiased causal assessment.

### 2.2. Genetic Variants Associated with PUD

GWAS and GWGAS involving 456,327 participants from UK Biobank indicated that 8 independent SNPs were strongly associated with PUD (*P* < 5 × 10^−8^; *r*^2^ < 0.1) [13]. Seven of 8 SNPs associated with PUD were included in the insomnia GWAS dataset and selected for the reverse causality analysis (Table 2).

### 2.3. Mendelian Randomization Analyses

Three Mendelian randomization analyses were carried out to explore the causal relationship between insomnia and PUD.

Firstly, conventional Mendelian randomization analyses, also called the inverse-variance weighted method, were implemented to regress the exposure (genetic variants in susceptibility to insomnia) against the outcome (genetic variants in susceptibility to PUD), with each variant as a data point.

Secondly, two sensitivity analyses were performed to evaluate the effect of pleiotropism on Mendelian random causality, relaxing the Mendelian randomization hypothesis partly. For instance, MR-Egger analysis is based on the InSIDE hypothesis, which requests that the size of any pleiotropic effect (from genetic variants to PUD, bypassing insomnia) should not be related to the size of the main effect (from genetic variants to insomnia). Another approach based on median assumes that when analyzing a large number of variants (some of which may be pleiotropic), these pleiotropies may well be material difference and therefore unlikely to be concentrated on a common median estimate. Conversely, effective variants without pleiotropy are more possibly to display more accordant and homogeneous effects (on insomnia and subsequent PUD), more possibly to cluster at the estimated median point. Causal reasoning is strengthened by the consistency of results among different approaches which propose s different hypothesis about pleiotropy; nevertheless, different results may suggest that some of the results were influenced by genetic pleiotropy.

Thirdly, in the case of a large number of pleiotropies, the genetic risk of PUD may also forecast the outcome of insomnia; we conducted reverse Mendelian randomization analysis using 8 SNPs associated with PUD (only 7 of 8 SNPs associated with PUD were included in the insomnia GWAS dataset and selected for the reverse causality analysis).

### 2.4. Functional Annotation

FUMA was used to functionally annotate; three gene mapping strategies were applied in FUMA to identify genes that are associated with the 242 SNPs, which were included in the MR analysis. In the positional mapping, we mapped 242 SNPs and SNPs in LD (*r*^2^ ≥ 0.6) with them to genes. The other two gene-mapping strategies were gene mapping based on eQTL and gene mapping based on chromatin interaction.

### 2.5. Pathway and Functional Enrichment Analyses

Enrichment analyses including GO (http://geneontology.org/) analysis and KEGG (https://www.kegg.jp/) analysis were carried out on David (https://david-d.ncifcrf.gov/).

### 2.6. Statistical Analyses

All SNPs were matched to the same effect allele among the different datasets. All statistical tests were 2-sided, and statistical significance was assessed by Bonferroni correction. All analyses were conducted in Stata (version 15), FUMA (version 1.2.4), and R (version 3.2.5).

## 3. Results

### 3.1. Causal Effect from Insomnia to PUD

**Figure 1:**
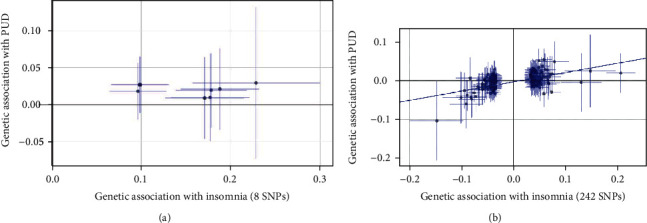
Comparison plot of the association between insomnia-related SNPs associated with insomnia and PUD: (a) 8 insomnia-related SNP analysis; (b) 242 insomnia-related SNP analysis. Each point displays the influence of the SNP on the insomnia and PUD. The slope of the line represents the causal association.

**Figure 2:**
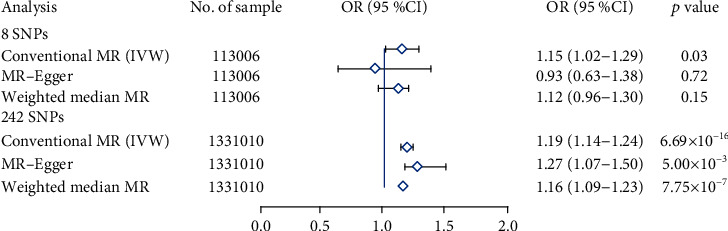
Forest plot estimates the effect of genetically increased insomnia risk on PUD. EA/NA: effect allele/no effect allele; OR: odds ratio; CI: confidence interval.

There is a first stage analysis using 8 SNPs that significant correlation to insomnia in the GWAS dataset (SSGAC) included 113,006 participants (*P* < 5.00 × 10^−8^, *r*^2^ < 0.1). All these 8 SNPs did not reach the Bonferroni-corrected significance level in the PUD GWAS (*P* > 0.006) (Table 1). The results in the conventional Mendelian randomization analysis showed that in a comparison of the effects of 8 SNPs on insomnia and PUD, the effects on insomnia and PUD were clearly consistent, indicating a significant causal relationship between insomnia and PUD (*P* = 0.03; OR, 1.15 (95% CI, 1.02-1.29)) (Figures 1 and 2). However, results were inconsistent between the other two methods, using the weighted median method (*P* = 0.15; OR, 1.12 (95% CI, 0.96-1.30)) and MR-Egger regression (*P* = 0.72; OR, 0.93 (95% CI, 0.63-1.38)), both with a wider CI (Figure 2). Those divergent results among those methods may indicate a bias of genetic pleiotropy towards these results.

Considering the limited number of SNPs and the limited sample size in this stage analysis which were insufficient to derive unbiased causal estimates, in order to make our sensitivity analysis to achieve sufficient statistical power, we retrieved 248 independent SNPs associated with insomnia from the most recent GWAS data involving 1,331,010 European people (*P* < 5.00 × 10^−8^; *r*^2^ < 0.1), which explained 2.6% of the variance in insomnia. 242 of 248 SNPs associated with insomnia were included in the PUD GWAS dataset, and all these 242 SNPs did not reach the Bonferroni-corrected significance level in the PUD GWAS (*P* < 0.0002) (see supplementary Table 1). The secondary set of analyses using 242 SNPs yielded consistent results. The conventional Mendelian randomization analysis indicated a causal relationship between insomnia and prevalent PUD; 1 SD increased insomnia incident was associated with a 19% higher risk of PUD (*P* = 6.69 × 10^−16^; OR, 1.19 (95% CI, 1.14-1.24)) (Figures 1 and 2).

Complementary analyses showed the consistent associations between insomnia and PUD through the weighted median method (*P* = 7.75 × 10^−7^; OR, 1.16 (95% CI, 1.09-1.23)) and MR-Egger regression method (*P* = 5.00 × 10^−3^; OR, 1.27 (95% CI, 1.07-1.50)) (Figure 2). There are no pleiotropy (MR-Egger intercept; *β* = −0.003, −0.001 to 0.004; *P* = 0.416) or heterogeneity between the Mendelian randomization estimates obtained for different SNPs (*I*^2^ = 9.8%, *P* = 0.0028 for heterogeneity). All the sensitivity analyses make it unlikely that pleiotropy would seriously affect our primary cause analysis. Causal reasoning is strengthened by the consistency of findings among diverse approaches that propose a different hypothesis about pleiotropy, which is consistent with the assumptions that pleiotropy was not responsible for this finding.

### 3.2. Causal Effect from PUD to Insomnia

**Figure 3:**
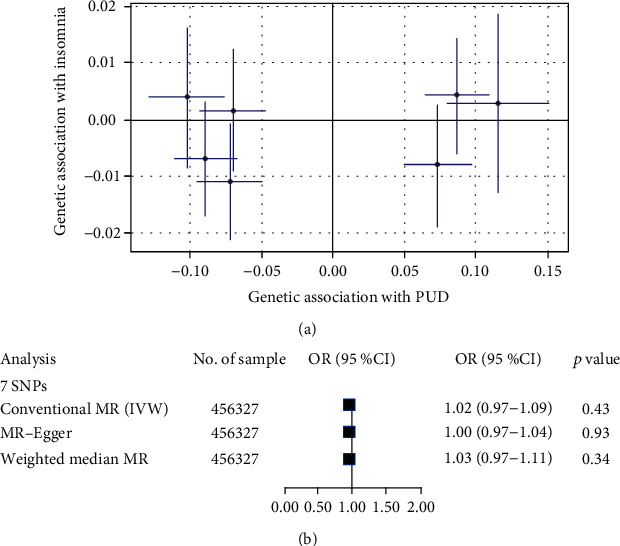
Causal effect from PUD to insomnia: (a) comparison plot of the association between PUD-related SNPs associated with insomnia and PUD; (b) forest plot estimates the effect of genetically increased PUD risk on insomnia. Each point displays the influence of the SNP on the insomnia and PUD.

Consider that PUD is also likely to be the causal factor of insomnia symptoms, reverse MR analysis was carried out using 7 SNPs associated with PUD, which were not associated with insomnia after Bonferroni corrected (*P* > 0.007) (Table 2). However, no evidence was found for the assumption that PUD event is related to insomnia outcomes in the conventional Mendelian randomization analysis (*P* = 0.43; OR, 1.02 (95% CI, 0.97-1.09)) (Figure 3). Results were unchanged after using the weighted median analysis (*P* = 0.34; OR, 1.03 (95% CI, 0.97-1.11)) and MR-Egger analysis (*P* = 0.93; OR, 1.00 (95% CI, 0.97-1.04)) (Figure 3). Those evidences indicated no reverse causality between PUD events and insomnia symptoms.

### 3.3. Functional Annotation

**Figure 4:**
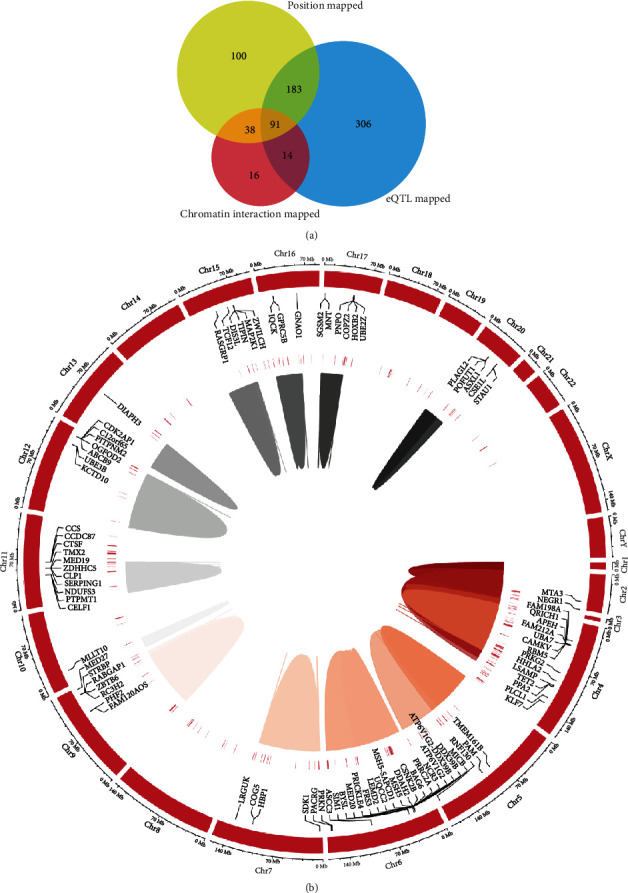
Genes mapped to insomnia-related SNPs: (a) venn diagram; (b) circos plot heatmap.

Three gene mapping methods were applied in FUMA to identify genes of interest on account of 242 SNPs included in the MR analysis. For mapping based on position, 412 genes were identified by mapping SNPs in the risk loci and in LD with the independent GAWS SNPs (*r*^2^ ≥ 0.6). Mapping based on eQTL implicated 594 genes and mapping based on chromatin interaction implicated 159 genes (Figure 4). A total of 748 genes were identified by the three methods; 91 of them were identified by all three methods, which were distributed on multiple chromosomes (see supplementary Table 2-4).

### 3.4. Gene Set Enrichment Analyses

**Figure 5:**
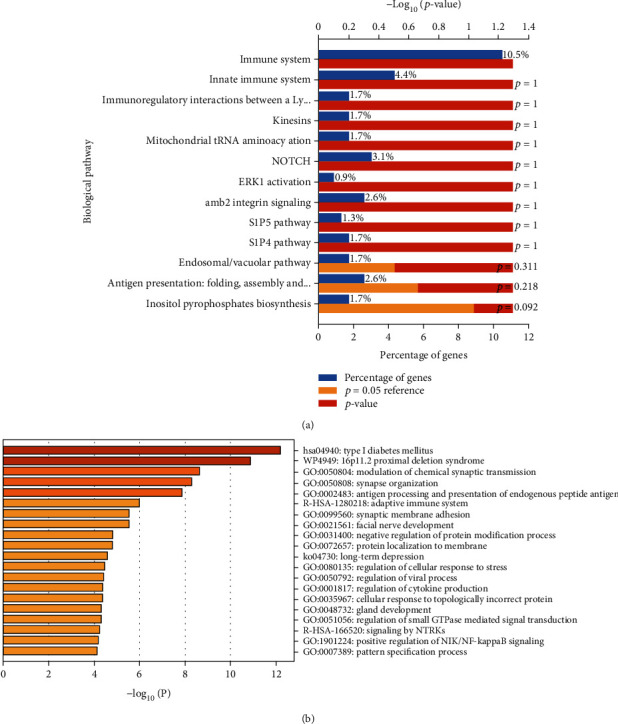
Enrichment analysis: (a) biological pathway of enrichment genes; (b) enrichment heatmap for selected GO.

**Figure 6:**
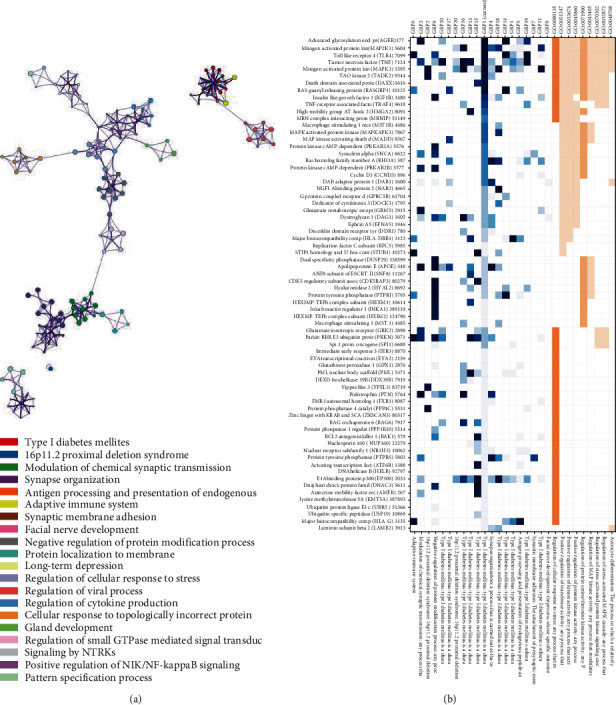
Gene cluster: (a) gene clusters and interactions between genes; (b) genes in the enrichment GO.

To investigate the possible mechanism of insomnia leading to PUD, all 748 genes identified by the methods above were used to conduct GO enrichment analysis at the online Functional Annotation Tool (DAVID). These genes were classified from three levels of molecular function, cellular component, and biological process to further understand their related functions. At the same time, the KEGG database was used for gene signaling pathway analysis to understand the signaling pathways involved in these genes. The results showed that in terms of cell components, the genes mainly focused on nucleus, cytoplasm, plasma membrane, etc. In terms of molecular function, the genes were mainly related to GTPase activity, MHC class I and MHC class II receptor activity, and transcription regulator activity. In terms of biological processes, genes are mainly related to the immune system, innate immune system, NOTCH, and mitochondrial tRNA aminoacylation (Figures 5 and 6). KEGG enrichment pathway analysis suggested that those genes were significant enrichment in the adaptive immune system, synaptic membrane adhesion, regulation of cellular response to stress, viral process and cytokine production, etc. (Figures 5 and 6).

## 4. Discussion

PUD is a chronic inflammatory disease; insomnia is closely related to gastroesophageal reflux and peptic ulcer [15]. Clinical studies have found that patients with poor sleep quality have a higher recurrence rate of PUD [8]. Despite observational studies indicate an association between insomnia and PUD, it is lacking of causal evidence for effects of insomnia on PUD. Both insomnia and PUD have a genetic predisposition. This causal study shows that insomnia is related to the increasing risk of PUD. The findings corroborate the results of several observational studies which suggested a positive correlation between insomnia and PUD risk.

Although the etiology of PUD is not yet clear, H. pylori infection, gastric acid overproduction, and weakened gastric mucosal protection mechanism are recognized as the main pathogenesis in recent years. Modern medicine suggests that abnormal sleep patterns may be a vital factor for the development of chronic inflammatory diseases. Abnormal sleep affects gastrointestinal function and is associated with worsening of functional gastrointestinal diseases. Sleep disturbances can increase the incidence of peptic ulcer. Sleep deprivation can lead to gastric mucosal erosion, which may involve the reduction of gastric mucosal blood flow, the suppression of cell proliferation, the influence of gastric mucosal repair, and the decrease of gastric mucosal ion barrier. The study found that after entering deep sleep, gastric mucous outflow, gastric mucosal blood flow, and melatonin secretion increased, whereas gastrin secretion decreased, which were helpful to prevent PUD development and recurrence. Recently, researchers found that the stomach and small intestine produce a chemical called TFF2 protein that repairs the ruptures at night, and lack of sleep reduces the production of this protein and increases the risk of stomach ulcers [16].

Insomnia is a neurological disease that can lead to complications in a variety of other systems. Insomnia can cause central nervous system and gastrointestinal function regulation imbalance, and long-term insomnia can also lead to anxiety and depression, which in turn can cause gastrointestinal nerve, endocrine, and immune disorders, which may produce somatization symptoms of the digestive system in different degrees. As stimulation of the vagus nerve can promote gastric acid secretion, vagotomy is commonly used to treat PUD clinically. The autonomic nervous system is closely related to the enteric nervous system. It is widely accepted that the enterobrain axis participates in the regulation of the alimentary canal and the maintaining of the intestinal immunity system through the mechanisms of intestinal permeability, intestinal endocrine signaling, and immune activation. But sleep disturbances or circadian rhythm disturbances may disrupt its normal function and may affect gastrointestinal sensitivity to ulcer preparations [17]. Further study suggests that melatonin may prevent PUD recurrence through clearing oxidative free radicals, promoting cell proliferation and gastric mucosal microcirculation, whereas sleep disorders cause a lack of melatonin, affecting the underlying protective effect of melatonin against PUD recurrence [18].

We conducted pathway-enrichment analysis for the genes associated with insomnia using gene clustering tools to explore the potential biological mechanisms between insomnia and PUD. We found that the subnetworks of the genes are enriched in multiple processes, including the immune system, regulation of viral process, facial nerve development, long-term depression, regulation of cytokine process, regulation of cellular response to stress, and mitochondrial tRNA aminoacylation. It is well-known that lacking sleep can lead to decreased immunity, so that the body including gastrointestinal is susceptible to bacteria or viruses. The interesting thing is that many genes involved in oxidative stress and mitochondrial function were found such as GPX1, DARS2, PRDX6, HAAO, RBM14-RBM4, NDUFS3, OGFOD2, and ATP5G1, suggesting insomnia may affect PUD through oxidative stress and mitochondrial function. Evidences suggest that reactive oxygen species (ROS) refer to the pathogenesis of PUD as well as possibly gastric cancer [19]. Sleep deprivation directly affects the oxidative state of the mucosa [20]. Recently, tests on fly and mouse have shown that sleep expropriation resulted in the amassment of ROS in the gut and consequent oxidative stress [21]. Alzoubi et al. reported that sleep deprivation increases oxidative stress as well as impairs learning and memory processes [22]. Similar with Reimund's hypothesis, sleep deprivation is an oxidative challenge, and adequate sleep may have a protective effect against oxidative damage [23]. Oxidative stress is thought to be a latent condition connected with insomnia. Systemic oxidative stress was increased, and serum antioxidant enzyme levels were decreased in insomnia patients in contrast with healthy people [24]. Mitochondrial respiratory complexes were reduced, and superoxide dismutase 2 (SOD2) was significantly increased in fatal familial insomnia (FFI) patient, suggesting that mitochondrial and protein synthesis mechanisms are reduced [25]. Mitochondrial damage increases and leads to an increase in oxidative stress [26]. Sleep disorders may promote the accumulation of neurotoxic proteins and oxidative stress [27].

Redox signal is involved in gastrointestinal physiology and pathophysiology. Redox signal regulates the Notch and Wnt signaling pathway principally by NADPH oxidase (NOx), thereby regulating the physiological self-renewal, propagation, transference, and differentiation of gastrointestinal epidermal cell. Broken redox homeostasis participates in the development and progression of diverse gastrointestinal diseases, like peptic ulcer, inflammatory bowel diseases, and colorectal cancer. Harmful “oxygen free radical overload” has serious impacts on intestinal mucosa and is relevant to the clinical outcome of inflammatory bowel disease [28, 29]. Oxidative stress is now recognized as an underlying causative factor and a key factor for the progress and severity of diseases, instead of the accompanying consequence of chronic gastrointestinal inflammations [30, 31]. Oxidative stress take part in the inflammatory reaction of H. pylori infection through a variety of redox signals. H. pylori infection results in the occurrence as well as the development of PUD, but the mechanism of it leading to various clinical manifestations is still unclear. During infection, changes in the balance of cell proliferation and apoptosis may lead to changes in the gastric mucosa. Existing studies reveal that H. pylori infection is closely relevant to an increasing synthesis of free radicals, which are thought as a major cause of cell death. Oxidative stress induced by H. pylori activates apoptosis through the intrinsic pathways. The alteration in structure and function of mitochondrial organelles caused by these processes are the fundamental causes of gastric mucosal toxicity and result in the occurrence of a variety of manifestations related to the infection. Accumulating evidences indicate that antioxidant therapy may contribute to the clinical therapies of patients with H. pylori infection. Many factors, including genetics, stress, and hygiene, take part in the pathogenic mechanisms of H. pylori infection. A link is found between H. pylori infection and increasing free radicals [32, 33]. The increasing of ROS in gastric mucosa in gastritis patients infected with H. pylori was considered to be related to bacterial load. As we all know, oxidative stress is one of the factors that lead to accelerated cell cycles and premature cell death, resulting in various degenerative and psychiatric diseases. From a physiological perspective, cell protects itself against oxidative stress through arousing antioxygen defense mechanisms referring to oxygen-scavenger enzymes like superoxide dismutase, catalase, and glutathione peroxidase [34]. Spirulina improves gastric ulcer induced by aspirin in albino mice through enhancing antioxidant and cell-protective defenses alleviating oxidative stress and inflammation [35].

In this study, we found that insomnia is an important risk factor for PUD, rather than a complication of PUD. The mechanism of insomnia effect on PUD may be through a variety of ways, such as the immune system; it is worth noting that oxidative stress might also take part in it. The advantages in this study are the design of the MR as well as the use of data from large-scale GWASs of insomnia and PUD, which provide sufficient statistical power. Another advantage is that the studies included in these MR analyses mainly included individuals of European ancestry, which reduces the bias attributable to demographic stratification. In addition, because genetic information is used as an instrumental variable, the method is less susceptible to confounder, and because common exposures (such as weight) do not negatively affect human genetic information (such as DNA sequence), this method is not affected by reverse causality.

This study may be limited because the validity of the results of MR studies may be threatened by pleiotropy. However, our results were consistent across all analyses based on different MR methods, and there was no indication of directional pleiotropy. Another drawback is that the MR study evaluated whether insomnia susceptibility is associated with PUD. However, our findings do not necessarily mean that insomnia itself is a cause of PUD. We cannot rule out that there are other causes of insomnia leading to PUD. In addition, the definition of insomnia includes a different set of chief complaints of insomnia. Another limitation is the overlap of individual segments included in GWASs for insomnia and PUD. This may introduce some bias in the MR estimates.

## 5. Conclusions

In conclusion, we found a significant association between insomnia and adverse PUD outcomes, highlighting the need to address sleep concerns as part of PUD management, and improving the quality of sleep might be a valuable way for populations suffering from insomnia to prevent PUD. Considering that patients with poor sleep quality are more prone to PUD recurrence, it is necessary to check up on these patients regularly. In addition, healthy people should also get enough sleep to prevent digestive problems.

## Figures and Tables

**Table 1:** Characteristics of the studied insomnia-associated SNPs in both insomnia and PUD GWAS.

SNP	Gene	EA	NA	Insomnia results			PUD results		
				BETA	SE	*P*	BETA	SE	*P*
rs375216017	MEIS1	GT	G	0.089574	0.015720	1.21*E* − 08	—	—	—
rs62144051	MEIS1	G	A	0.096151	0.016318	3.81*E* − 09	0.018165	0.019416	0.35
rs62144053	MEIS1	A	G	0.098931	0.016272	1.20*E* − 09	0.026932	0.019223	0.16
rs62144054	MEIS1	A	G	0.097846	0.016237	1.68*E* − 09	0.026976	0.019221	0.16
rs113851554	MEIS1	T	G	0.178239	0.020371	2.14*E* − 18	0.019566	0.025498	0.44
rs182588061	MEIS1	T	G	0.228449	0.036320	3.18*E* − 10	0.029322	0.051821	0.57
rs139775539	MEIS1	A	AC	0.186994	0.022403	7.00*E* − 17	—	—	—
rs11679120	MEIS1	A	G	0.187813	0.022461	6.18*E* − 17	0.021005	0.027931	0.45
rs115087496	MEIS1	C	G	0.177042	0.022576	4.43*E* − 15	0.009710	0.029911	0.73
rs549771308	MEIS1	C	CT	0.090546	0.015777	9.51*E* − 09	—	—	—
rs11693221	MEIS1	T	C	0.170821	0.022572	3.79*E* − 14	0.009066	0.028027	0.75
rs574753165	SCFD2	G	A	0.394740	0.067503	4.98*E* − 09	—	—	—

PUD: peptic ulcer disease; EA/NA: effect allele/no effect allele; BETA: effect size; SE: standard error of the effect size; *P*: *P* values indicate genome-wide significance in GWAS.

**Table 2:** Characteristics of the studied PUD-associated SNPs in both insomnia and PUD GWAS.

SNP	Gene	EA	NA	PUD results		Insomnia results			
				BETA	SE	*P*	BETA	SE	*P*
rs681343	FUT2	C	T	-0.088770	0.011169	1.9*E* − 15	-0.006920	0.005108	0.18
rs2976388	PSCA	G	A	0.086730	0.011318	1.8*E* − 14	0.004309	0.005152	0.40
rs10500661	CCKBR	T	C	-0.101570	0.013439	4.1*E* − 14	0.004008	0.006322	0.53
rs147048677	MUC1	C	T	-0.152180	0.022310	9.0*E* − 12	—	—	—
rs78459074	MUC6	A	G	0.115348	0.018241	2.6*E* − 10	0.002804	0.008030	0.73
rs34074411	GAST	C	T	-0.072050	0.011397	2.6*E* − 10	-0.010940	0.005242	0.04
rs687621	ABO	A	G	0.073539	0.012116	1.3*E* − 09	-0.007970	0.005474	0.13
rs9581957	CDX2	C	T	-0.069730	0.011819	3.6*E* − 09	0.001601	0.005474	0.77

PUD: peptic ulcer disease; EA/NA: effect allele/no effect allele; BETA: effect size; SE: standard error of the effect size; *P*: *P* values indicate genome-wide significance in GWAS.

## Data Availability

All data are available on reasonable request from the corresponding author.

## Supplementary Materials

Supplementary Materials Supplementary Table 1: insomnia-associated SNPs. Supplementary Table 2: position mapping genes. Supplementary Table 3: eQTL mapping genes. Supplementary Table 4: chromatin interaction mapping genes.
